# Supplementary material for: Comparative multiomics analysis of cell physiological state after culture in a basket bioreactor
Source: Sci Rep. 2022 Nov 23;12:20161. doi: 10.1038/s41598-022-24687-4 (PMC9686226; doi:10.1038/s41598-022-24687-4)
Supplement: Supplementary file 1 — Supplementary Information 1. [file 41598_2022_24687_MOESM1_ESM.zip › raw data/Proteomic raw data/2.func.anno/2.3.KEGG/KEGG.anno.pdf]

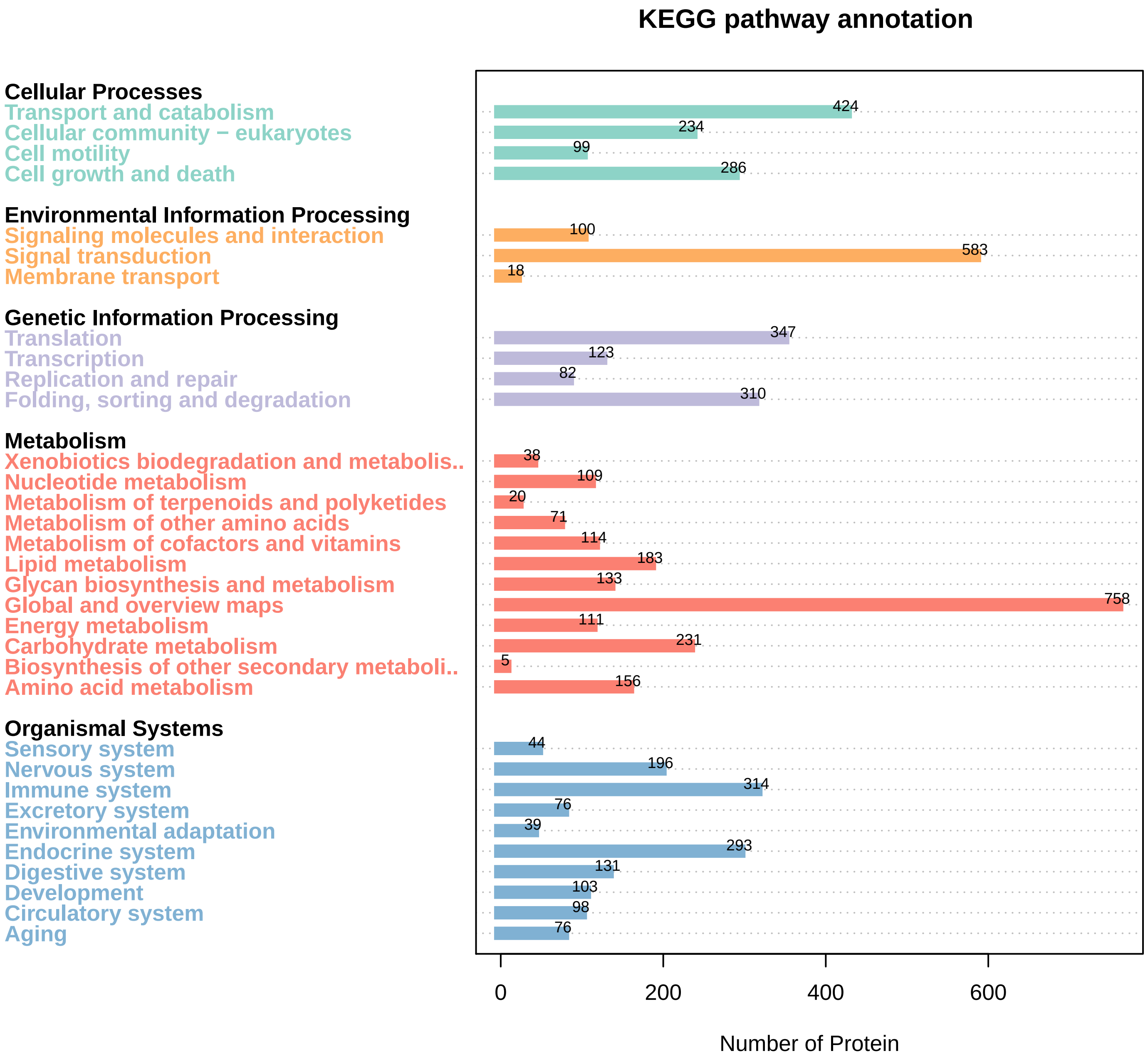
KEGG pathway annotation
Cellular Processes
Transport and catabolism
Cellular community − eukaryotes
Cell motility
Cell growth and death
Environmental Information Processing
Signaling molecules and interaction
Signal transduction
Membrane transport
Genetic Information Processing
Translation
Transcription
Replication and repair
Folding, sorting and degradation
Metabolism
Xenobiotics biodegradation and metabolis..
Nucleotide metabolism
Metabolism of terpenoids and polyketides
Metabolism of other amino acids
Metabolism of cofactors and vitamins
Lipid metabolism
Glycan biosynthesis and metabolism
Global and overview maps
Energy metabolism
Carbohydrate metabolism
Biosynthesis of other secondary metaboli..
Amino acid metabolism
Organismal Systems
Sensory system
Nervous system
Immune system
Excretory system
Environmental adaptation
Endocrine system
Digestive system
Development
Circulatory system
Aging
424
234
99
286
100
583
18
347
123
82
310
38
109
20
71
114
183
133
758
111
231
5
156
44
196
314
76
39
293
131
103
98
76
0
200
400
600
Number of Protein
